# In ovo threonine administration and early feeding strategies modulate oxidative stress responses and liver metabolism in ducks subjected to post-hatch feed deprivation

**DOI:** 10.3389/fvets.2026.1891766

**Published:** 2026-08-04

**Authors:** V. Ács, Nóra Katalin Szeli, A. Tischler, O. Csötönyi, I. Jócsák, I. Benedek, J. Nagy, Szilvia Áprily, Ö. Petneházy, J. Turbók, J. Enyezdi, V. Halas

**Affiliations:** 1 Hungarian University of Agriculture and Life Sciences (Kaposvár Campus), Kaposvár, Hungary; 2 AVI-VET Ltd., Kaposvár, Hungary; 3 Medicopus Nonprofit Ltd., Kaposvár, Hungary; 4 Animal Health Diagnostic Department, Animal Health Diagnostic Directorate, National Food Chain Safety Office, Kaposvár, Hungary; 5 Pathodiagnostica Ltd., Pécs, Hungary

**Keywords:** antioxidant status, gene expression, in-ovo, leukocyte, threonine

## Abstract

Early post-hatch development represents a critical physiological period during which nutritional interventions may influence metabolic adaptation, oxidative balance, and tissue development in poultry. This study investigated the effects of *in ovo* threonine (Thr) administration and post-hatch Hydrogel® supplementation, with or without Thr, on growth performance, oxidative status, antioxidant-related gene expression, hematological parameters, and liver histology in ducks subjected to delayed feed access. A total of 606 fertilized duck eggs were randomly assigned to seven experimental treatments, including immediately fed controls and groups exposed to a 36 h post-hatch feed deprivation period. Relative expression of Nrf2 and GPX1 was evaluated at hatch and after feed deprivation using quantitative PCR. Growth performance, FRAP, MDA, leukocyte profiles, and liver histopathology were also assessed. Delayed feed access significantly reduced early growth performance (*p* < 0.05), whereas partial compensatory growth was observed later in the rearing period. Oxidative status was also significantly affected, as reflected by altered FRAP and MDA values (*p* < 0.05). The highest antioxidant capacity was observed in the immediately fed saline-injected control and in ovo threonine-treated groups, while increased lipid peroxidation was detected in several delayed-fed treatments. Nrf2 expression generally decreased after feed deprivation, whereas GPX1 expression showed a more variable response pattern among treatments and sampling times. Histological evaluation revealed significant temporal changes in hepatic fat accumulation, hydropic degeneration, and lymphocytic infiltration (*p* < 0.05). Feed deprivation was associated with increased inflammatory and degenerative liver alterations at later sampling points. Overall, delayed post-hatch feeding induced marked oxidative and metabolic disturbances in ducks, while early nutritional interventions only partially alleviated the negative physiological effects of feed deprivation.

## 1 Introduction

Poultry meat is one of the most widely consumed and economically important protein sources worldwide. Among poultry species, duck meat is valued for its favorable nutritional composition, particularly its high content of polyunsaturated fatty acids and essential amino acids (1). Despite these advantages, duck meat is highly susceptible to oxidative damage, and newly hatched ducklings are frequently exposed to environmental and management stressors that disrupt redox balance. One of the most critical early-life challenges is delayed access to feed. Under commercial conditions, post-hatch fasting may extend up to 72 h, impairing gastrointestinal development, digestive capacity, and muscle protein deposition (2, 3). High stocking density and other environmental stressors may further increase oxidative stress through elevated reactive oxygen species (ROS) production, thereby compromising growth performance, immune function, and physiological stability (4). Consequently, nutritional strategies aimed at improving antioxidant capacity and immune competence have become increasingly important in modern poultry production systems. Previous studies demonstrated that dietary composition markedly influences oxidative status, lipid stability, and immune responses in poultry (5–7).

Functional amino acids have recently gained considerable attention because of their roles in intestinal development, metabolic regulation, oxidative balance, and carcass quality (8, 9). Threonine (Thr), the third limiting amino acid in corn–soybean meal diets (10, 11), is essential for maintaining mucosal barrier integrity by supporting goblet cell density and mucin synthesis (12). In addition, Thr has been associated with regulation of intestinal gene expression, antioxidant status, and intestinal barrier function (13, 14). Previous studies reported that dietary Thr supplementation may improve immune responses, antioxidant capacity, and productive performance in poultry exposed to physiological or environmental stress (15–18). Elevated dietary Thr levels have also been associated with improved productive performance, whereas Thr deficiency may contribute to hepatic triglyceride accumulation and altered lipid metabolism (19, 20). Early post-hatch development represents a physiologically sensitive transition during which birds shift from yolk-derived nutrients to exogenous feed sources. Delayed access to feed disrupts this transition and may impair intestinal maturation, metabolic adaptation, and early growth performance (8, 21, 22). Moreover, early nutritional stress may induce persistent physiological and metabolic alterations that remain detectable later in development (4). Oxidative imbalance is considered a major component of this early-life challenge. Antioxidant defense systems rely partly on transcription factors such as Nrf2, which regulate several antioxidant enzymes, including superoxide dismutase (SOD) and glutathione peroxidase (GPX) (23–25). In particular, GPX1 plays a central role in neutralizing hydro-peroxides and maintaining cellular redox homeostasis (26). In ducks, oxidative stress is closely associated with hepatic lipid metabolism and inflammatory responses, while Nrf2 signaling is increasingly recognized as an important regulator of hepatic metabolic adaptation (27–29). To mitigate early-life nutritional stress, both in ovo nutrient administration and early post-hatch nutritional supplementation have been investigated in poultry production systems. In ovo feeding provides targeted nutritional support during embryogenesis and may improve intestinal development, digestive capacity, and post-hatch adaptation (2, 30). Similarly, Hydrogel®-based supplementation -which is a common method in practice- may provide hydration and nutrients during temporary feed deprivation immediately after hatch. However, limited information is available regarding the effects of threonine delivered either in ovo or through Hydrogel® supplementation in ducks subjected to early feed deprivation. For this research, Thr was selected because of its fundamental role in intestinal integrity, stimulating mucin synthesis, and supporting antioxidant and immune functions during early development. Hydrogel®—as a commercially available and applied early feeding method to provide water and readily available nutrients—was offered during the post-hatch fasting period, with the aim of partially compensating for the negative effects of delayed access to feed.

Therefore, the present study aimed to compare two complementary early nutritional strategies—a prenatal intervention (in ovo threonine administration) and an immediate post-hatch intervention (Hydrogel® supplementation)—as potential approaches to mitigate the adverse effects of delayed access to feed on growth performance, oxidative status, antioxidant-related gene expression (NRF2 and GPX1), hematological parameters, and liver morphology in ducks subjected to early post-hatch feed deprivation.

## 2 Materials and methods

### 2.1 Experimental design and management

The experiment was conducted at the Department of Farm Animal Nutrition, Hungarian University of Agriculture and Life Sciences (MATE), Kaposvár Campus. All procedures followed the guidelines of the Hungarian National Scientific Ethical Committee of Animal Experimentation (protocol license number: SO/31/00444-2/2021, KA-3232) and complied with Directive 2010/63/EU on the protection of animals used for scientific purposes. All animal handling, housing, sampling, and experimental interventions were performed in accordance with institutional standards for the care and use of experimental animals.

For tissue collection, birds were euthanized by carbon dioxide (CO₂) inhalation using a gradual-fill euthanasia chamber. Ducklings were exposed to increasing CO_2_ concentrations until loss of consciousness was achieved, followed by confirmation of death prior to necropsy and tissue sampling. The procedure was carried out in accordance with Directive 2010/63/EU and the approved experimental protocol.

A total of 630 fertilized Cherry Valley commercial (meat-type) duck eggs were obtained for the experiment. Prior to incubation 24 eggs were excluded during tray placement because they did not meet the required quality criteria, resulting in 606 eggs being set for incubation. The eggs were stored for 6 days at 20 °C without turning or additional humidification. Incubation was performed in a PLM 1350- type setter and PLM1350-type hatcher two-stage incubators equipped with an automated monitoring system for temperature, humidity, and ventilation. Incubation conditions followed the recommendations of Cherry Valley (31) according to manufacturer’s recommendations (see Table 1).

**Table 1:** Incubation parameters for duck eggs.

Hatching phase	Days of incubation	Temperature (°C)	Rel. humidity (%)	Turning	Ventilation (vent. %)	Cooling (at 20 °C room temperature)	Cooling time (min)	Spraying (with warm water)	Candling
Setter phase	1	37.8	70	Every 2 h, 45 °-45 ° angle	0	None	None	None	None
	2				0				
	3				0				
	4		55		25	Air ventilation (by opening the incubator door) once daily	3–5 min	After cooling, spray lightly with warm water (mist-like)	
	5				25				
	6				25				
	7				25				
	8				25				
	9				50				
	10				50		5–7 min		
	11				50				
	12				50				
	13				50				
	14	37.5	55		75	Until the beginning of pipping: twice daily (with door opening/outside the machine) using air ventilation	2 × 10 min	After cooling, spray with a larger amount of warm water compared to the previous phase	×
	15				75				None
	16				75				
	17				75		2 × 15 min		
	18				75				
	19				75				
	20	37.2			100		2 × 20 min	After cooling, apply intensive spraying until pipping	
	21				100				
	22				100				
	23				100				
	24				100				
	25				100				
Egg transfer to hatcher + in ovo treatment	25	37.2	60 (until intensive pipping)	None	75	None	None	None	×
Hatching phase	26	37.2			75–100				none
	27	37.0	75–80						
	28	37.0							

Egg candling was performed on days 14 and 25 of incubation to identify infertile eggs and dead embryos, which were subsequently removed from the incubator. Fertility rate and hatching rate were calculated according to the following equations:

$$\text{Fertility rate}\;\;(\%)={\frac{\text{Number of fertile eggs}}{\text{Total number of eggs}\;\mathrm{set}}}^{*}100$$

$$\text{Hatching rate for eggs}\;\mathrm{set}\;\;(\%)=\frac{\text{Number of hatched ducklings}}{\text{Total number of eggs}\;\mathrm{set}}\times 100$$

$$\begin{array}{l} \text{Hatching rate for fertile eggs}\;\;(\%)\\ =\frac{\text{Number of hatched ducklings}}{\text{Total number of fertile eggs}}\times 100 \end{array}$$

The in ovo treatment procedure was based on the method described by Uni and Ferket (30), with the modification that the injection site was sealed using a sterile elastic patch instead of paraffin wax. Elastic sealing materials have also been successfully applied in poultry incubation studies to prevent excessive moisture loss and contamination while maintaining satisfactory hatchability (32). This stage was selected because the embryo routinely ingests the amniotic fluid immediately before hatch, allowing efficient uptake of the administered solution. Prior to injection, treatment solutions were pre-warmed to 30 °C. Eggs were disinfected with iodine, and a small opening was drilled into the air chamber at the blunt end of the egg. All injections were carried out under sterile conditions in a laminar airflow cabinet (ScanLaf, LaboGen Inc., Denmark). Embryo position was verified before injection to minimize the risk of embryonic injury. Each egg received 0.5 mL of treatment solution administered into the amniotic fluid. Treatments consisted of physiological saline (0.9% NaCl) or L-threonine (≥98.5% purity, Sigma-Aldrich, St. Louis, MO, United States) dissolved in physiological saline (0.5% threonine). Following the injection, the opening was sealed with a sterile elastic patch to prevent contamination and excessive moisture loss before the eggs were returned to the incubator.

Ducklings were sexed immediately after hatch by cloacal examination and individually weighed before allocation to the experimental groups. Birds were then allocated to the treatments while maintaining a balanced sex ratio across groups. Each treatment consisted of 72 ducklings (36 females and 36 males), which were randomly assigned to eight replicate deep-litter floor pens containing nine birds each. Environmental conditions and management were maintained according to Cherry Valley recommendations (2017). Seven experimental treatments were established to evaluate the effects of delayed post-hatch feeding and different early threonine supplementation strategies. Two groups with immediate access to feed served as positive controls. In group Int_0, no *in ovo* treatment was applied, whereas eggs in group IoS_0 received an *in ovo* saline injection on day 25 of incubation. All remaining groups were subjected to a 36 h post-hatch feed deprivation period to model commercially relevant early-life nutritional stress. Under commercial hatchery and transport conditions, newly hatched poultry may experience delayed access to feed and water for 24–48 h due to hatch window variation, handling, and transport logistics (22). Therefore, a 36 h deprivation period was selected as a representative but non-extreme challenge model, allowing the evaluation of early nutritional interventions while minimizing excessive mortality and severe physiological impairment.

To evaluate strategies aimed at alleviating the adverse effects of delayed feeding, nutritional interventions were applied exclusively to feed-deprived ducklings. Accordingly, no in ovo treatment or early nutritional supplementation was applied in group Int_36. Eggs in group IoS_36 received an in ovo injection of physiological saline (0.9% NaCl), whereas eggs in group IoT_36 received threonine dissolved in physiological saline. Both solutions were administered into the amniotic fluid. In addition, two groups received *ad libitum* post-hatch gel supplementation during the feed deprivation period. Birds in group Int_G36 received a commercially available gel-based supplement (Hydrogel®, Bábolna Feed Ltd.; Table 2), while birds in group Int_GT36 received threonine-enriched Hydrogel® supplementation during the same period. A summary of the experimental treatments is presented in Table 3.

**Table 2:** Composition of hydrogel® as given by the producer.

Parameter	Unit	Value
DM	%	5
ME	MJ/kg dry matter	5.4
Corn starch	%	30
Vitamin C	mg/kg	5,000
*Pediococcus acidilactici* (E1712)	CFU/g	1 × 1,010

*DM, dry matter; ME, metabolizable energy.

**Table 3:** Treatment groups and experimental design.

Treatment	Feed access	Early feeding
Int_0	Immediately after hatch	—
InS_0		*In ovo*-saline
Int_36	Delayed (36 h) solid feed access	—
InS_36		*In ovo*-saline
InT_36		*In ovo*-Thr
Int_G36		Hydrogel
Int_GT36		Hydrogel + Thr

Throughout the experiment, birds had ad libitum access to feed and water. A starter diet in crumbled form was provided between 1 and 21 days of age, followed by a grower diet in pelleted form from day 22 to day 42. Experimental diets were produced at the Department of Farm Animal Nutrition and formulated on a corn–soybean meal basis according to Cherry Valley breeder recommendations (33). Nutrient composition, including dry matter, crude protein, crude fat, ash, calcium, and phosphorus content, was analyzed at the University Laboratory Center of MATE according to AOAC methods (34).

The ingredients and calculated nutrient content of the feed are shown in Table 4.

**Table 4:** Ingredients and analyzed nutrient contents of the basal diets (g/kg) of cherry valley ducks.

Ingredients	Starter (1–21)	Grower (22–42)
Corn	246	291
Barley	227	—
Wheat	—	375
Soybean meal (44.2%)	437	274
Fat, vegetable	48	19
MCP	16.2	16.2
Limestone	14	14
NaCl	4.7	4.1
DL-metionine	1.6	1.2
L-treonine	0.5	0.5
Premix^a^	5.00	5.00
Total	1000.00	1000.00

Nutrient content (g/kg)	Starter (1–21)	Grower (22–42)
AMEn (MJ/kg)	12.23	12.56
Crude protein	220.9	165.5
Crude fat	63.6	32.3
Crude fiber	32.00	24.6
Lysine	13.4	9.3
M + C	8.8	6.8
Treonine	9.6	7
Tryptophan	3.1	2.3
Ca	9	8.7
P_available_	7.5	7
P_non-phytic_	4.5	4.4
Na	1.7	1.6

a Premix feed contents per kilogram: Zn: 22032 mg, Cu:3200 mg, Fe: 160.20 mg, Mn: 21948 mg, I: 300 mg, Se: 70 mg, Co: 20 mg, Vit. A: 3240000 IU, D3: 810000 IU, Vit. E: 20800 mg, Vit. K3: 810 mg, Vit. B1: 810 mg, Vit. B2: 1890 mg, Vit. B3: 10800 mg, Vit. B5: 3240 mg, Vit. B6: 1350 mg, Vit B12: 6.8 mg, Folic acid: 270 mg, Biotin: 32 mg.

Live body weight was recorded at hatch (Day 1), at 36 h post-hatch, and on Days 21 and 42. Feed intake (FI) was measured on a pen basis for each experimental period by recording the amount of feed offered and the residual feed remaining in the feeders. Feed conversion ratio (FCR) was calculated per pen. At each sampling point, blood, liver, and jejunum samples were collected for further analyses.

### 2.2 Methods for determining antioxidant activity and lipid peroxidation

#### 2.2.1 Malondialdehyde (MDA)

Lipid peroxidation was evaluated by measuring malondialdehyde (MDA) levels according to the method of Heath and Packer (35) with minor modifications. Plasma samples were centrifuged at 3,760 rpm for 12 min at 4 °C, and the supernatant was mixed with a reagent containing 20% (w/v) trichloroacetic acid and 0.5% (w/v) thiobarbituric acid. The reaction mixture was incubated at 96 °C for 30 min and subsequently cooled on ice. After centrifugation at 10,000 rpm for 5 min, absorbance was measured at 532 and 600 nm using a spectrophotometer (BioRad SmartSpec Plus). MDA concentration was calculated by correcting nonspecific absorbance at 600 nm from total absorbance at 532 nm using an extinction coefficient of 156 mM^−1^·cm^−1^ and expressed as nmol·g^−1^ fresh weight (FW).

#### 2.2.2 FRAP assay

Total antioxidant capacity was determined using the ferric reducing antioxidant power (FRAP) assay according to Benzie and Strain (36) with minor modifications. Briefly, 100 μL of plasma sample was mixed with 3.0 mL of freshly prepared FRAP reagent, and absorbance was measured at 593 nm immediately after mixing and again after 4 min of incubation at 37 °C using a spectrophotometer. A standard curve was prepared using ascorbic acid (100–1,000 μM), and results were expressed as μmol ascorbic acid equivalents per gram fresh weight (μmol·AAE·g^−1^·FW).

### 2.3 Gene expression

Four ducks per treatment group were euthanized immediately after hatch and following the 36 h feed deprivation period for molecular analyses. Liver and jejunum samples (~3 × 3 mm) were collected and preserved in RNAlater® (Thermo Fisher Scientific, Waltham, MA, United States) until further processing. Total RNA was extracted using the RNeasy® Mini Kit (QIAGEN Sciences, Hilden, Germany) according to the manufacturer’s instructions. Tissue homogenization was performed in RLT buffer supplemented with 1% *β*-mercaptoethanol using a TissueLyser II system (QIAGEN Sciences, Hilden, Germany). RNA concentration and purity were determined spectrophotometrically using a NanoDrop™ One instrument (Thermo Fisher Scientific, Waltham, MA, United States). Only RNA samples with A260/A280 ratios between 1.8 and 2.1 and A260/A230 ratios above 1.8 were included in the analyses. For cDNA synthesis, 100 ng of total RNA was reverse-transcribed using the QuantiTect® Reverse Transcription Kit (QIAGEN Sciences, Hilden, Germany) according to the manufacturer’s protocol. Relative expression of GPX1 and Nrf2 genes was determined by quantitative PCR using GAPDH as the reference gene. Primer sequences were adopted from Yarru et al. (37) and are presented in Table 5.

**Table 5:** Primer design.

Primer	Sequence
Gpx-1	For-5′CAGTACATCATCTGGTCGCC3′
	Rev-5′CCTGGATCTTGATGGTTTCG3′
Nrf-2	For-5′GTTGAATCATCTGCCTGTGG3′
	Rev-5′TAAGCTAGGTGGTCGAGTGC3′
GAPDH	For-5′ATGTTCGTGATGGGTGTGAA3′
	Rev-5′CTGTCTTCGTGTGTGGCTGT3′

Gene expression data obtained from qPCR analyses were evaluated according to the method of Livak and Schmittgen (38). Expression of the target genes (*GPX1* and *Nrf2*) was normalized to the housekeeping gene *GAPDH*, which was assumed to remain stable across treatments (39). ΔCt values were calculated as follows:

$$\mathrm{\Delta Ct}=Ct_{\text{target}}-Ct_{\text{reference}}$$

Relative expression levels were then calculated using efficiency-corrected fold change (FC) values (40)

$$\text{ΔΔCt}=\mathrm{\Delta C}t_{\text{sample}}-\bar{\mathrm{\Delta C}t_{\text{control}}}$$

Fold change (FC) values were calculated as:

$$\mathrm{FC}=E^{-\text{ΔΔCt}}$$

where *E* represents gene-specific amplification efficiency.

### 2.4 Statistical analysis

Statistical analyses were performed using R software (RStudio, (41)) with the packages *dplyr*, *tidyr*, *ggplot2*, *boot* (42), *patchwork*, and *multcompView*.

Data were analyzed according to the experimental design. Pen was considered the experimental unit for feed intake and feed conversion ratio analyses, whereas individual birds served as experimental units for body weight, gene expression, blood, and histological measurements.

Prior to statistical analysis, data were tested for normality and homogeneity of variance using Levene’s test. Hatch weight data were analyzed using one-way ANOVA to evaluate the effects of *in ovo* treatments, and results are presented as least square means with pooled RMSE values. Growth performance parameters, including live weight (LW) and average daily gain (ADG), were analyzed using two-way ANOVA with treatment and sex as fixed effects, including their interaction according to the following:

$$Y_{\mathrm{ij}}=\mu +T_{i}+S_{j}+{\left(T\times S\right)}_{\mathrm{ij}}+e_{\mathrm{ij}}$$

where *Y* is the observed trait, *μ* is the overall mean, *T* is the fixed effect of treatment, *S* is the fixed effect of sex, (*T* × *S*) is the interaction between treatment and sex, and *e* is the residual error.

Feed intake (FI), feed conversion ratio (FCR) antioxidant, hematological, and other biochemical parameters were evaluated using one-way ANOVA.

$$Y_{i}=\mu +T_{i}+e_{i}$$

where treatment was considered the fixed effect.

When significant treatment effects were detected, means were separated using Tukey’s *post hoc* test. Differences were considered significant at *p* < 0.05.

Relative gene expression was calculated according to Livak and Schmittgen (38) using the efficiency-corrected equation described by Rao et al. (40):

$$\mathrm{FC}=\frac{E_{\text{target}}^{-\Delta {\mathrm{Ct}}_{\text{target}}}}{E_{\text{reference}}^{-\Delta {\mathrm{Ct}}_{\text{reference}}}}$$

Fold change (FC) values were log_2_-transformed (log_2_FC) to improve data symmetry and facilitate the interpretation of up- and downregulation patterns.

$$\log_{2}\mathrm{FC}=\log_{2}(\mathrm{FC})$$

Due to the relatively small sample size, bootstrap resampling (5,000 iterations) with bias-corrected and accelerated (BC_a_) confidence interval was applied to log_2_FC values (43). Differences in gene expression among treatments were evaluated using linear models.

Histological alterations were evaluated semi-quantitatively by microscopic examination performed by the same observer, blinded to the treatment groups, using predefined scoring criteria based on lesion severity. Lesions were scored on a four-point ordinal scale (0–3), where 0 indicated the absence of the lesion and scores of 1, 2, and 3 represented mild, moderate, and severe alterations, respectively. For the statistical evaluation of lesion frequencies, scores were further dichotomized into absence (score 0) or presence (scores 1–3). Associations between treatment group, sampling time, and the frequency of each histological alteration were evaluated using Pearson’s Chi-square test. Fisher’s exact test was applied when the expected cell frequency in any contingency table was <5. Statistical significance was accepted at *p* < 0.05.

## 3 Results

### 3.1 Hatchability results

Following candling procedures, 575 viable eggs (94.88%) remained for further incubation. Before hatch, treatment groups were standardized to equal egg numbers (*n* = 83 per treatment). During the remaining incubation period, 3 embryos (0.5%) were lost due to mortality or handling damage, while 17 embryos (2.96%) died during hatching.

After hatch, 51 ducklings were classified as non-viable or unsuitable for further rearing, resulting in 504 first-grade ducklings. Embryonic losses and hatch-related mortality were evenly distributed among treatment groups, and no treatment-associated pattern was observed. Overall hatchability was 83.17% based on total eggs set and 84.56% based on fertile eggs. No significant differences in hatchability were observed among treatment groups (*p* = 0.60), and sex had no significant effect on hatchability (*p* = 0.26).

### 3.2 Live weight and average daily gain

No significant differences were observed in hatch weight among the experimental groups (*p* = 0.56), indicating that neither *in ovo* saline nor threonine administration affected body weight at hatch (see Table 6).

**Table 6:** Effects of the in-ovo saline and threonine on the live weight.

Treatments	Intact				*In ovo* saline		*In ovo* threonine
	Int_0	Int_36	Int_G36	Int_GT36	IoS_0	IoS_36	IoT_36
Hatching weight	**58.59**				**58.72**		**58.08**
*p*-value	0.56						
RMSE	0.57						

*Cont, intact control; Del_Int, delay-fed intact; Hyd and HydT, immediate Hydrogel® supplement after hatch with/without Thr; IoS, in ovo saline; IoT, *in ovo* threonine. Bold values indicate statistically significant differences (*p* < 0.05).

Live weight was significantly affected by treatment at both 36 h post-hatch (*p* < 0.001) and day 21 (*p* = 0.001), whereas no treatment × sex interaction was detected at any sampling point. At 36 h post-hatch, all delayed-fed groups showed significantly lower body weight compared to the immediately fed control groups, regardless of the applied supplementation strategy. Similar differences were observed on day 21, where the Int_36, IoS_36, and IoT_36 groups remained lighter than the Int_0 and Int_G36 treatments. By day 42, treatment effects were still significant (*p* = 0.013), although differences among groups became less pronounced. Male ducks showed higher live weight than females at day 42 (*p* = 0.004), whereas sex had no significant effect at earlier sampling points (see Table 7).

**Table 7:** Liveweight of the birds after feed deprivation and on days 21 and 42.

Day of measurement	LW2 (g)							LW21 (g)						
Treatment	Int_0	IoS_0	Int_36	IoS_36	IoT_36	Int_G36	Int_GT36	Int_0	IoS_0	Int_36	IoS_36	IoT_36	Int_G36	Int_GT36
Female	71.51	70.7	51.7	54.8	53	54.4	53.9	1665.8	1641.5	1548.9	1547.2	1551.8	1585.3	1564
Male	71.65	69.2	52	53.2	52.4	54.6	53.8	1666.3	1608.3	1574.9	1583.4	1562.1	1702.8	1600.4
Effect of sex	0.31							0.08						
Effect of treatment	**<0.001**							**0.001**						
Tukey test	**a**	**a**	**b**	**b**	**b**	**b**	**b**	**a**	**ab**	**b**	**b**	**b**	**a**	**ab**
Interaction	0.7							0.86						

Day of measurement	LW42 (g)						
Treatment	Int_0	IoS_0	Int_36	IoS_36	IoT_36	Int_G36	Int_GT36
Female	3657.3	3733	3598.1	3693.2	3691.5	3692.2	3774.1
Male	3777	3900	3628.9	3828.8	3845.9	3935.5	3813.3
Effect of sex	**0.004**						
Effect of treatment	**0.013**						
Tukey test	**ab**	**a**	**b**	**ab**	**ab**	**a**	**ab**
Interaction	0.68						

*Int_0, intact group with immediate feed access; IoS_0, *in ovo* saline with immediate feed access; Int_36, intact group with 36 h feed deprivation; IoT_36, *in ovo* threonine with 36 h feed deprivation; Int_G36, intact group with Hydrogel® supplement in the transport boxes during the 36 h feed deprivation; Int_GT36, intact group with Hydrogel® and threonine. Bold values indicate statistically significant differences (*p* < 0.05).

Average daily gain (ADG) was significantly influenced by treatment during all evaluated periods (*p* < 0.01) (Table 8). During the first 36 h post-hatch (ADG 0–2), delayed-fed groups exhibited negative growth rates as a consequence of feed deprivation, while immediately fed control groups showed positive weight gain (*p* < 0.001). Similar treatment-related differences were observed during the 0–21 day period, where delayed-fed groups generally showed lower ADG values compared to immediately fed birds. Between days 22 and 41, partial compensatory growth was observed in several delayed-fed groups, resulting in higher ADG values in some supplemented treatments compared to the intact control group (*p* = 0.01). Overall ADG between Days 0 and 41 also differed among treatments (*p* = 0.01). Sex significantly affected ADG during the 0–21 and 22–41 day periods, as well as across the entire experiment, with males showing higher growth rates than females. No treatment × sex interaction was detected.

**Table 8:** Average daily gain of birds in the examined period.

Day of measurement	ADG 0–2							ADG 0–21						
Treatment	Int_0	IoS_0	Int_36	IoS_36	IoT_36	Int_G36	Int_GT36	Int_0	IoS_0	Int_36	IoS_36	IoT_36	Int_G36	Int_GT36
Female	12.62	13.3	−5	−5.6	−5.3	−5.3	−4.9	71.51	75.6	71.1	70.6	70.8	72.3	71.6
Male	11.85	11	−5.2	−5.6	−5.4	−4.8	−4.9	76.48	74.9	72.3	72.9	71.6	78.3	73.4
Effect of sex	0.19							**0.03**						
Effect of treatment	**<0.001**							**<0.001**						
Tukey test	**a**	**a**	**b**	**b**	**b**	**b**	**b**	**b**	**c**	**a**	**a**	**a**	**bc**	**bc**
Interaction	0.16							0.32						

Day of measurement	ADG 22–41							ADG 0–41						
Treatment	Int_0	IoS_0	Int_36	IoS_36	IoT_36	Int_G36	Int_GT36	Int_0	IoS_0	Int_36	IoS_36	IoT_36	Int_G36	Int_GT36
Female	99.1	104.6	101.9	107.2	106.7	104.5	108.6	48.5	51.4	49.7	52.3	52.1	50.9	52.9
Male	103.7	113	102.6	112.5	113.6	109.5	111.1	50.9	55.5	50.3	54.9	55.4	53.4	54.2
Effect of sex	**0.0063**							**0.0053**						
Effect of treatment	**0.01**							**0.01**						
Tukey test	**a**	**ab**	**a**	**b**	**c**	**ab**	**c**	**a**	**b**	**a**	**b**	**b**	**a**	**b**
Interaction	0.91							0.88						

*Int_0, intact group with immediate feed access; IoS_0, *in ovo* saline with immediate feed access; Int_36, intact group with 36 h feed deprivation; IoT_36, *in ovo* threonine with 36 h feed deprivation; Int_G36, intact group with Hydrogel® supplement in the transport boxes during the 36 h feed deprivation; Int_GT36, intact group with Hydrogel® and threonine. Bold values indicate statistically significant differences (*p* < 0.05).

Feed intake (FI) during the first 36 h post-hatch and the 0–21 day period was affected by treatment (*p* < 0.001 and *p* = 0.0068, respectively). During the deprivation period, no feed consumption was recorded in the delayed-fed groups, whereas the immediately fed controls consumed comparable amounts of feed. Lower FI values were still observed in delayed-fed birds during the 0–21 day period. In contrast, treatment had no significant effect on FI between days 22 and 41 or over the entire experimental period.

Feed conversion ratio (FCR) was influenced by treatment only during the first 36 h post-hatch (*p* < 0.001), corresponding to the lack of feed intake in the deprived groups. No further differences were detected during the subsequent growth phases (see Table 9).

**Table 9:** Effect of different early feeding methods on feed intake and feed conversion ratio.

Treatment	FI 0–2	FI 0–21	FI 22–41	FI 0–41	FCR 0–2	FCR 1–21	FCR 22–41	FCR 0–41
Int_0	5.4a	100.8a	271.6	177.5	0.42a	1.32	2.72	2.05
IoS_0	5.4a	100.7a	267.6	175	0.52a	1.35	2.51	1.97
Int_36	0b	96.6b	269	174.9	0b	1.35	2.65	2.05
IoS_36	0b	94.6b	257.6	167.7	0b	1.32	2.37	1.91
IoT_36	0b	93.7b	270.4	172	0b	1.4	2.4	1.9
Int_G36	0b	94.6b	270.2	175.3	0b	1.29	2.6	1.96
Int_GT36	0b	93.7b	264.4	170.3	0b	1.33	2.44	1.94
*p*-value	**<0.001**	**0.0068**	0.52	0.29	**<0.001**	0.41	0.07	0.13
RMSE	0.48	2.99	10.35	5.92	0.06	0.06	0.17	0.78

*Int_0, intact group with immediate feed access; IoS_0, *in ovo* saline with immediate feed access; Int_36, intact group with 36 h feed deprivation; IoT_36, *in ovo* threonine with 36 h feed deprivation; Int_G36, intact group with Hydrogel® supplement in the transport boxes during the 36 h feed deprivation; Int_GT36, intact group with Hydrogel® and threonine. Values with different superscript letters (a, b) within the same column differ significantly (*p* < 0.05).

Although delayed feed access markedly reduced early body weight, partial compensatory growth was observed in the Hydrogel®-supplemented groups by day 21. By the end of the experiment, only the intact delayed-fed group (Int_36) remained significantly lighter than the immediately fed controls. Male ducks also reached higher final body weight compared to females.

### 3.3 Hematological parameters

**Figure 1:**
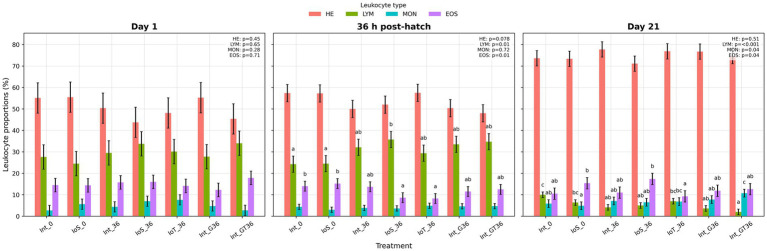
Hematology results at different timepoints. *HE, heterophyls; LYM, lymphocytes; MON, monocytes; EOS, eosinophils; Int_0, intact group with immediate feed access; IoS_0, in ovo saline with immediate feed access; Int_36, intact group with 36-hour feed deprivation; IoT_36, in ovo threonine with 36-hour feed deprivation; Int_G36, intact group with Hydrogel® supplement in the transport boxes during the 36 h feed deprivation; Int_GT36, intact group with Hydrogel® and threonine.

The effects of early feeding strategies and threonine supplementation on leukocyte subpopulations are presented in Figure 1. Proportions of heterophils (HE), lymphocytes (LYM), monocytes (MON), and eosinophils (EOS) were evaluated at hatch (day 1), at 36 h post-hatch, and on day 21 in order to characterize hematological responses associated with delayed feed access and early nutritional interventions.

No significant treatment effects were observed at day 1 for heterophils (*p* = 0.45), lymphocytes (*p* = 0.65), monocytes (*p* = 0.28), or eosinophils (*p* = 0.71). Heterophil proportions ranged between 43.7 and 55.5%, while lymphocyte values varied between 24.5 and 34.0% across treatments. Monocyte and eosinophil proportions also showed only limited variation among groups. At 36 h post-hatch, treatment significantly influenced lymphocyte (*p* = 0.01) and eosinophil (*p* = 0.01) proportions, whereas heterophil values showed a tendency toward significance (*p* = 0.078). Delayed-fed treatments generally tended to exhibit higher lymphocyte proportions compared with the immediately fed control groups. In contrast, eosinophil proportions were lower in the Int_36 and IoT_36 treatments compared to the remaining groups. By day 21, significant treatment effects were detected for lymphocytes (*p* < 0.001), monocytes (*p* = 0.04), and eosinophils (*p* = 0.04). Lymphocyte proportions declined markedly across all treatments, ranging from 2.0% in Int_GT36 to 10.0% in Int_0. Monocyte values were highest in the Int_GT36 and Int_G36 groups, while lower levels were observed in the immediately fed controls. Eosinophil proportions also differed among treatments, with elevated values observed in IoS_36, whereas lower proportions were generally detected in several delayed-fed groups.

### 3.4 Antioxidant capacity and lipid peroxidation

**Figure 2:**
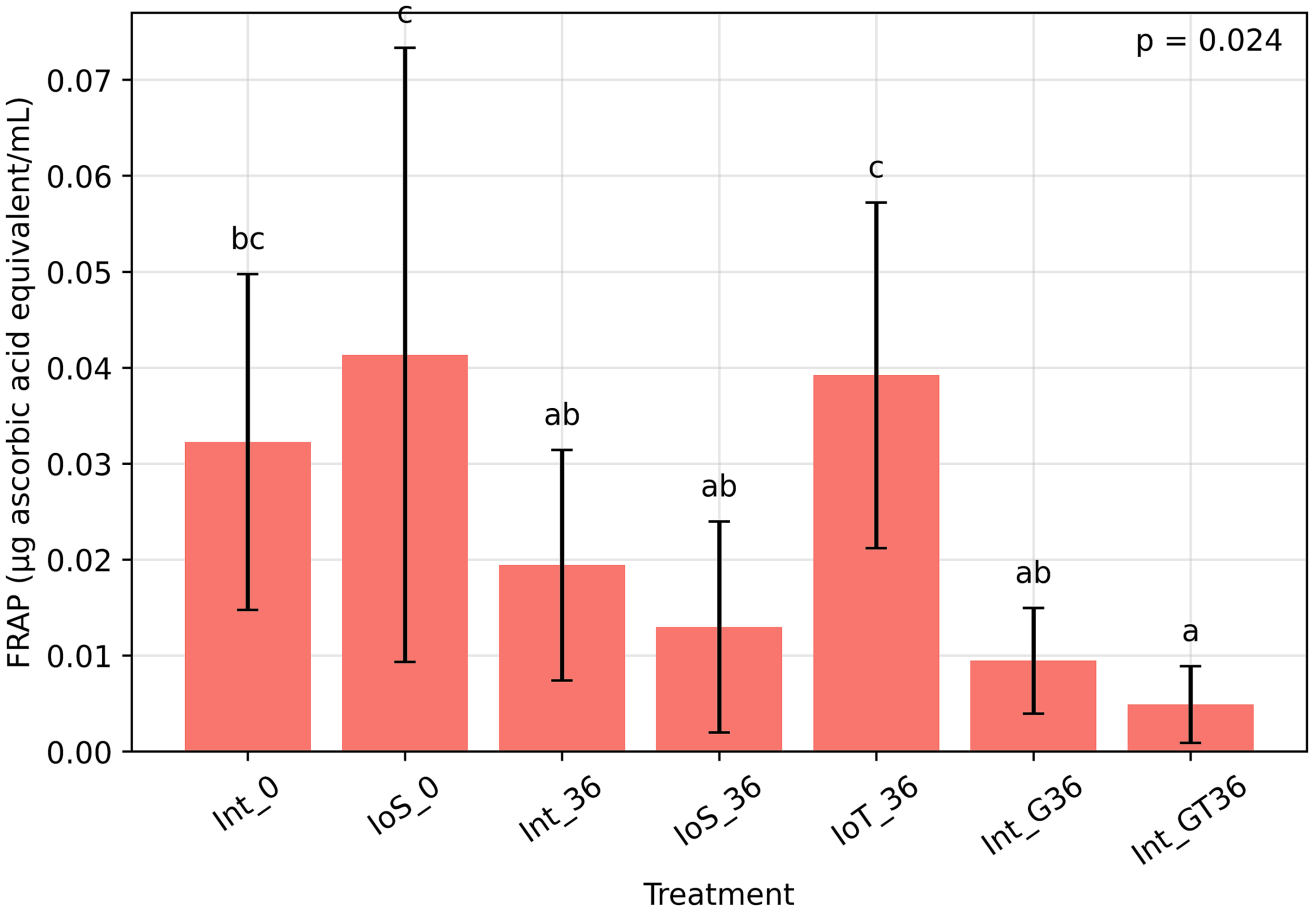
Total antioxidant capacity of plasma (FRAP) on day 21. *Int_0, intact group with immediate feed access; IoS_0, in ovo saline with immediate feed access; Int_36, intact group with 36-hour feed deprivation; IoT_36, in ovo threonine with 36-hour feed deprivation; Int_G36, intact group with Hydrogel® supplement in the transport boxes during the 36 h feed deprivation; Int_GT36, intact group with Hydrogel® and threonine.

The effects of delayed feed access and different early nutritional interventions on plasma total antioxidant capacity are presented in Figure 2. FRAP values were determined on day 21 in order to evaluate the antioxidant status of ducks subjected to the different feeding strategies.

FRAP values differed significantly among treatments (*p* < 0.05). The highest antioxidant capacity was observed in the immediately fed saline-treated control group (IoS_0; 0.041 μg ascorbic acid equivalents/mL) and in the *in ovo* threonine group (IoT_36; 0.039 μg/mL), both showing significantly higher values than the Int_GT36 group (0.005 μg/mL). Intermediate FRAP values were detected in the Int_0, Int_36, IoS_36, and Int_G36 groups. The lowest antioxidant capacity was observed in the Int_GT36 treatment.

**Figure 3:**
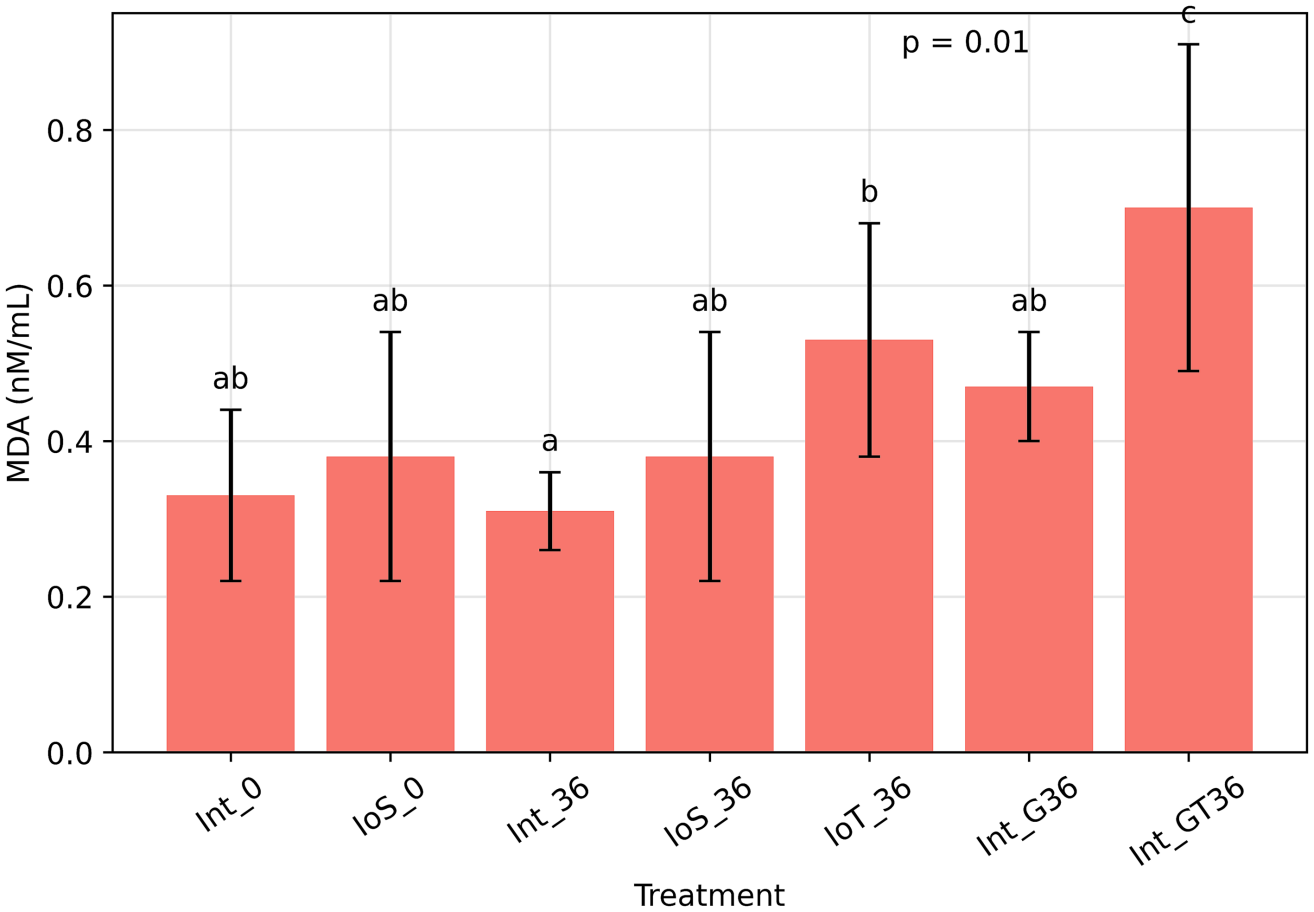
Plasma malondialdehyde (MDA) concentrations on day 21 in ducks subjected to different early feeding treatments. *Int_0, intact group with immediate feed access; IoS_0, in ovo saline with immediate feed access; Int_36, intact group with 36-hour feed deprivation; IoT_36, in ovo threonine with 36 h feed deprivation; Int_G36, intact group with Hydrogel® supplement in the transport boxes during the 36 h feed deprivation; Int_GT36, intact group with Hydrogel® and threonine.

The MDA levels on day 21 are summarized in Figure 3.

MDA levels were significantly affected by treatment (*p* < 0.05). The highest lipid peroxidation was observed in the Int_GT36 group (0.70 nM/mL), which showed significantly higher values compared to the other treatments. Relatively high MDA concentrations were also detected in the IoT_36 (0.53 nM/mL) and Int_G36 (0.47 nM/mL) groups. In contrast, lower MDA levels were measured in the Int_0, IoS_0, Int_36, and IoS_36 treatments, with the lowest value observed in Int_36 (0.31 nM/mL).

### 3.5 Gene expression results

**Figure 4:**
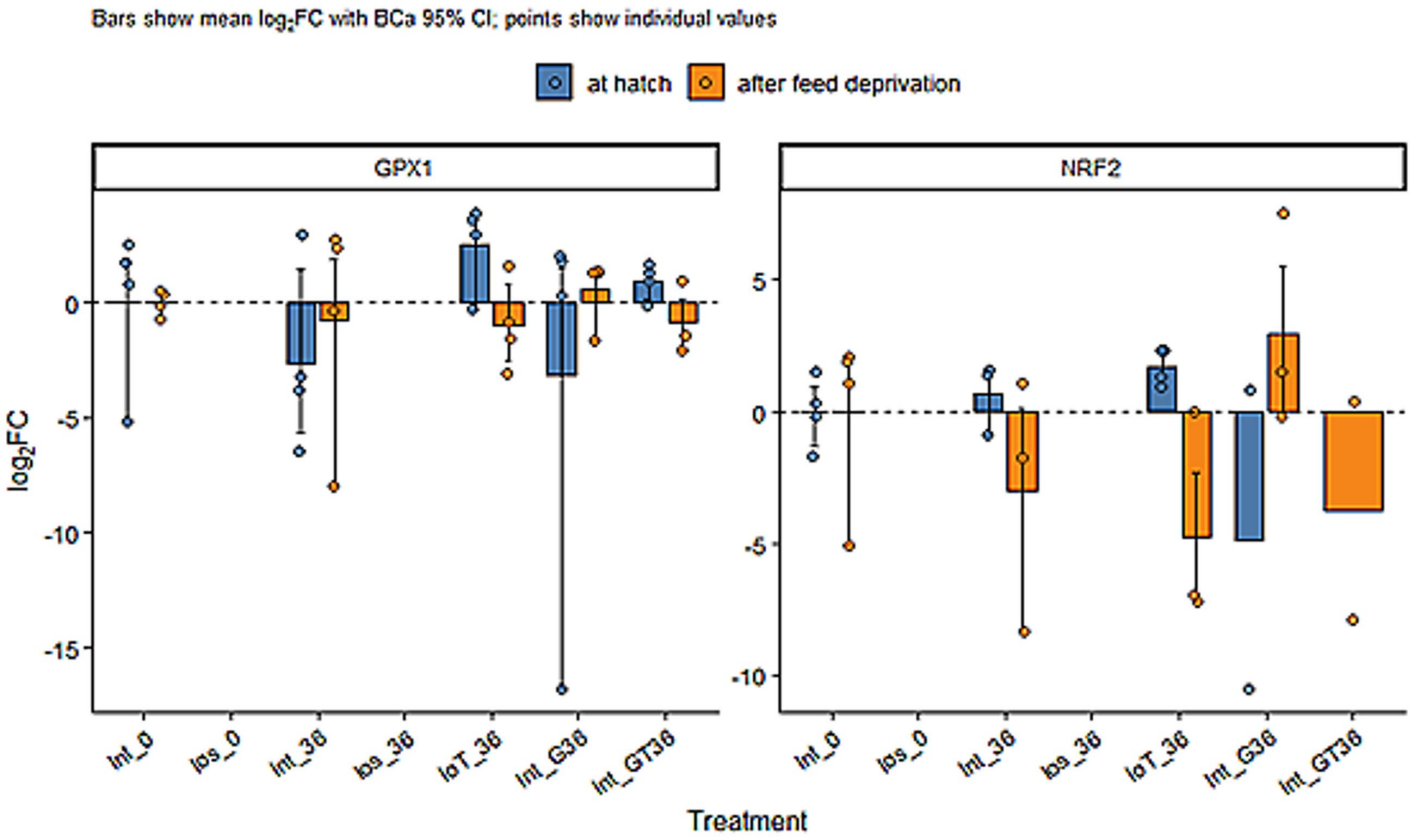
Relative expression of the examined genes (GPX1 and Nrf2) compared to the housekeeping gene (GAPDH). *Int_0, intact group with immediate feed access; IoS_0, in ovo saline with immediate feed access; Int_36, intact group with 36 h feed deprivation; IoT_36, in ovo threonine with 36 h feed deprivation; Int_G36, intact group with Hydrogel® supplement in the transport boxes during the 36 h feed deprivation; Int_GT36, intact group with Hydrogel® and threonine.

Relative expression of GPX1 and Nrf2 was evaluated at hatch and after the 36 h post-hatch feed deprivation period. Expression values were normalized to the housekeeping gene (GAPDH) and subsequently expressed as log_2_ fold change relative to the Int_0 control group (see Figure 4).

GPX1 expression showed considerable variability among treatments and sampling time points. At hatch, the Int_36 group generally exhibited negative log_2_FC values, indicating lower expression relative to the control group, although marked individual variation was also present. In contrast, the IoT_36 treatment showed the highest positive expression values, suggesting increased GPX1 expression following *in ovo* threonine supplementation. The Hydrogel®-supplemented groups displayed more moderate expression patterns, but substantial dispersion among individual samples was still observed.

After the 36 h feed deprivation period, GPX1 expression patterns changed in several treatments. In the Int_36 group, expression values shifted closer to baseline levels, indicating less pronounced downregulation compared to hatch day. Expression in the IoT_36 treatment also declined after deprivation, with several samples approaching or falling slightly below the control level. The Int_G36 group showed particularly high variability after deprivation, including some strongly negative values. As expected, expression values in the Int_0 control group remained around zero at both sampling points, although moderate biological variation was still present. Overall, GPX1 expression appeared to be influenced by both treatment and sampling time, with the clearest responses observed in the Int_36 and IoT_36 groups.

Compared to GPX1, Nrf2 showed a more distinct temporal response. At hatch, most treatment groups exhibited positive log_2_FC values relative to the control group, indicating generally increased Nrf2 expression during the immediate post-hatch period. The highest mean expression values were observed in the IoT_36 treatment, while the Int_36 group also showed positive regulation relative to the intact control birds.

Following the feed deprivation period, Nrf2 expression decreased in several treatments. In the Int_36 group, mean log_2_FC values shifted below zero, indicating downregulation relative to the control after fasting. A similar decline was observed in the IoT_36 group, although substantial variation among individual samples remained present. The Hydrogel®-supplemented groups also tended to show predominantly negative expression values after deprivation. Overall, the results suggest that Nrf2 expression declined following feed deprivation, whereas GPX1 responses appeared more moderate and variable among treatments.

### 3.6 Liver histopathology and fat deposition

**Figure 5:**
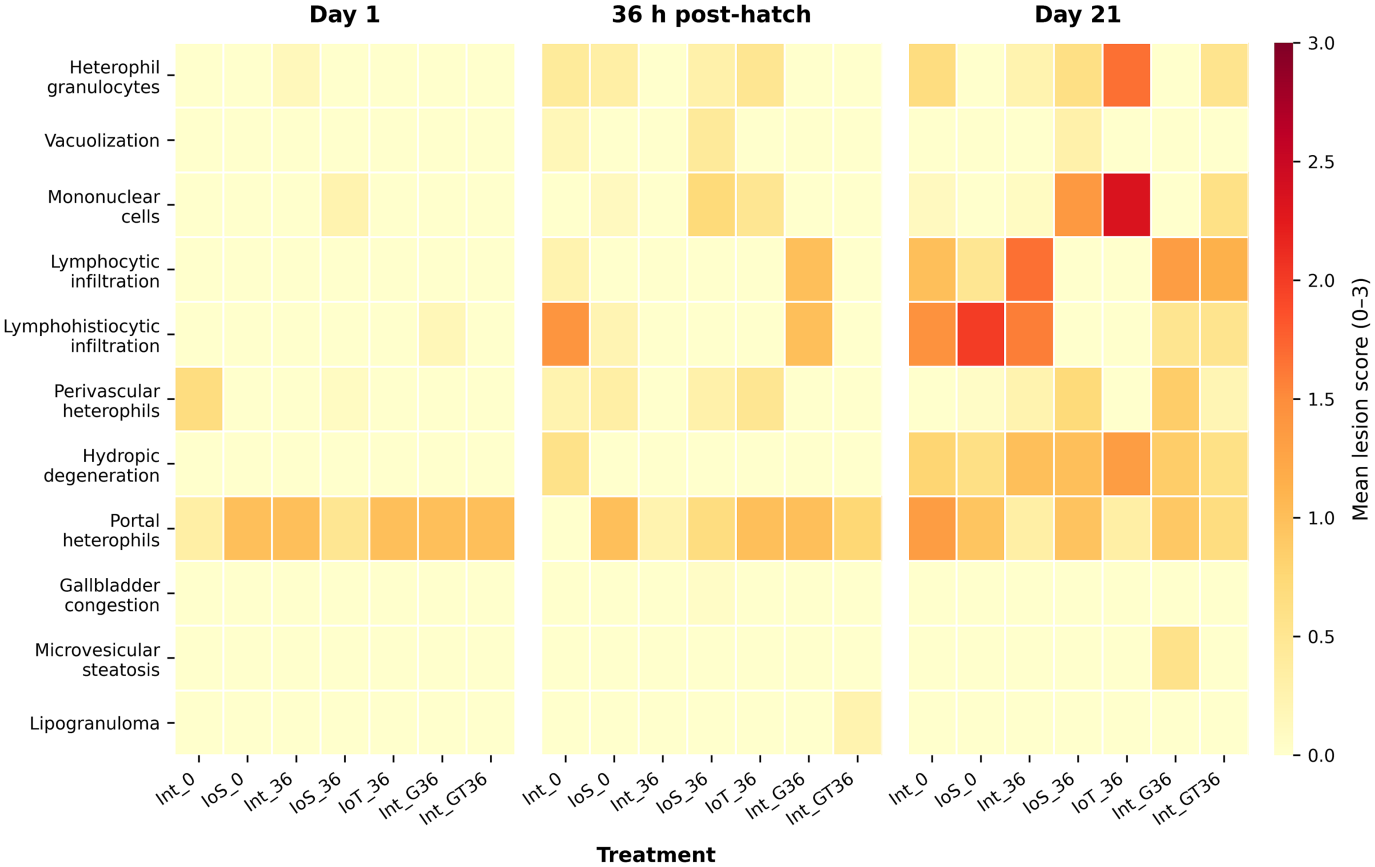
Heatmap visualization of semi-quantitative hepatic lesion scores across treatments and sampling times. Color intensity represents the mean severity scores of the evaluated histopathological alterations, with darker colors indicating more pronounced lesions. The heatmap illustrates overall lesion distribution patterns and does not directly represent statistical significance.

Semi-quantitative evaluation of liver lesions revealed marked treatment- and age-related histopathological alterations (Figure 5). In the heatmap, lesion severity is represented by a color gradient based on the mean histological scores of the evaluated parameters, where darker colors indicate more pronounced alterations. The heatmap is intended to visualize overall lesion distribution patterns among treatments and sampling times rather than direct statistical significance.

**Figure 6:**
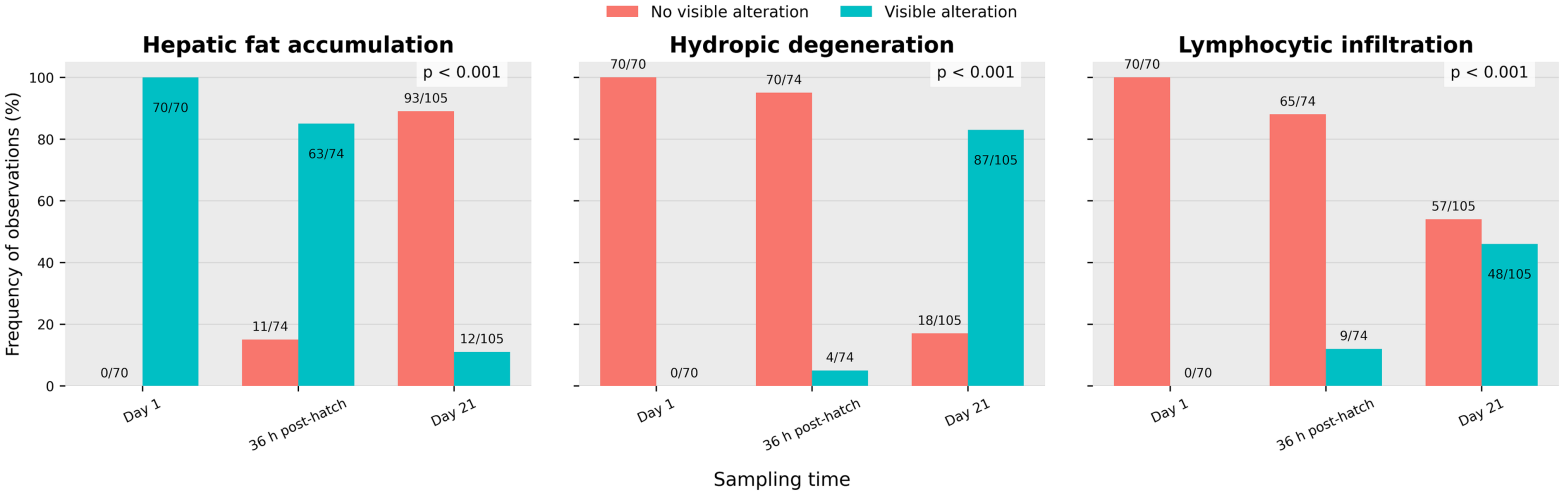
Frequency of hepatic histopathological alterations across sampling times.

At hatch, hepatic vacuolization and fat accumulation were more pronounced in several treatment groups, particularly in the immediately fed control groups. Following the 36 h feed deprivation period, increased hydropic degeneration and inflammatory cell infiltration became more evident in delayed-fed treatments, especially in the IoS_36 and Int_G36 groups. By Day 21, mononuclear and heterophil cell infiltration remained elevated in several delayed-fed groups, whereas vacuolization scores generally decreased compared with earlier sampling points. Overall, the heatmap analysis suggested that early feed deprivation induced persistent hepatic alterations associated with inflammatory and degenerative changes, although the severity and distribution of lesions varied among treatments and sampling times (see Figure 6).

At hatch, hepatic vacuolization and fat accumulation were more pronounced in several treatment groups, particularly in the immediately fed control groups. Following the 36 h feed deprivation period, increased hydropic degeneration and inflammatory cell infiltration were observed in delayed-fed treatments, especially in the IoS_36 and Int_G36 groups. By day 21, mononuclear and heterophil cell infiltration remained elevated in several delayed-fed groups, whereas vacuolization scores generally decreased compared to earlier sampling points. Overall, the heatmap analysis indicated that early feed deprivation was associated with persistent hepatic alterations, although the severity and distribution of lesions varied among treatments and sampling times.

## 4 Discussion

The present study demonstrates that delayed access to feed during the first 36 h post-hatch may influence early growth performance, oxidative status, antioxidant-related gene expression, and liver morphology in ducks. As the immediate post-hatch period represents a highly sensitive physiological transition when birds shift from yolk-derived nutrients to exogenous feed intake, this stage seems particularly vulnerable to nutritional disturbances and oxidative imbalance (8, 22). In agreement with previous poultry studies, hatch weight was not affected by the treatments, indicating that embryonic development itself remained largely unaffected. However, noticeable differences emerged after the feed deprivation period, as all delayed-fed groups exhibited reduced live weight and early weight loss compared with the immediately fed controls (Int_0 and IoS_0). Similar responses have been described in broiler chickens, where delayed access to feed impaired intestinal development, nutrient utilization, and early growth performance (2, 21). These findings suggest that delayed feeding primarily affects post-hatch metabolic adaptation rather than embryonic growth itself. Partial compensatory growth was also observed later in the rearing period, as early growth depression was not fully recovered in all treatments, particularly in the intact delayed-fed group (Int_36). Previous studies demonstrated that nutritional stress during the immediate post-hatch period may induce persistent physiological and metabolic alterations detectable later in life, including impaired intestinal maturation and altered metabolic regulation (4, 44). In the present experiment, neither Hydrogel® supplementation nor *in ovo* Thr administration fully counteracted the negative effects of delayed feeding on growth performance, emphasizing the biological importance of immediate nutrient availability after hatch. Hydrogel® supplementation partially influenced several physiological and molecular parameters but failed to restore growth performance to the level of immediately fed ducklings. This suggests that although gel-based supplements may provide hydration and limited nutrient availability during the fasting period, they cannot fully substitute for the complex nutritional and metabolic stimuli provided by complete feed. Similar conclusions have been reported in studies evaluating alternative early feeding strategies in poultry, where immediate access to balanced feed remained superior to nutritional supplements alone (3, 22).

The oxidative stress parameters further reflected the effects of early nutritional challenge. Significant differences in FRAP and MDA values demonstrated that both antioxidant capacity and lipid peroxidation were influenced by the treatments. Interestingly, the elevated FRAP values observed not only in the *in ovo* threonine-treated group but also in the saline-injected control group may suggest that early hydration itself contributed to improved antioxidant status during the immediate post-hatch period. Hatching is associated with intense metabolic activity, increased oxygen exposure, and elevated ROS generation, which may promote transient oxidative stress during the transition from embryonic to post-hatch life. Therefore, improved hydration during late embryogenesis may partially support redox homeostasis and antioxidant defense mechanisms during this physiologically demanding period. In contrast, the Hydrogel®-supplemented groups, particularly Int_GT36, showed markedly lower FRAP values. At the same time, elevated MDA concentrations were detected in the IoT_36, Int_G36, and Int_GT36 groups, indicating increased lipid peroxidation despite activation of antioxidant defense pathways. Similar responses have been reported in poultry exposed to nutritional or environmental stress, where enhanced antioxidant activity occurred simultaneously with elevated oxidative damage markers and lipid peroxidation (4, 13, 48). Increased MDA concentrations are widely considered indicators of oxidative membrane damage associated with excessive ROS production and disturbed redox balance (7, 35). The simultaneous increase in antioxidant capacity (FRAP) and lipid peroxidation (MDA) may initially appear contradictory. However, this pattern likely reflects a compensatory physiological response to oxidative challenge. Increased ROS production can stimulate antioxidant defense mechanisms while oxidative damage is still occurring, particularly during acute stress. Consequently, elevated FRAP values do not necessarily indicate complete protection against oxidative damage but rather activation of endogenous antioxidant systems in response to increased oxidative pressure (4, 45). Similar oxidative responses have been described in broilers exposed to environmental stressors, where increased hepatic MDA concentrations and altered antioxidant enzyme activity occurred together with disturbances in Nrf2-related signaling pathways (24). The gene expression results further support the occurrence of oxidative and metabolic adaptation following early nutritional stress. Nrf2 expression was generally higher at hatch, particularly in the IoT_36 group, whereas expression became more variable and tended to decrease after the 36 h feed deprivation period in several delayed-fed treatments, including Int_36 and Int_GT36. As Nrf2 is a key transcription factor regulating cellular antioxidant defense, these changes may reflect alterations in the regulation of oxidative stress responses during the transition from endogenous yolk utilization to exogenous nutrition. However, because only mRNA expression was determined, these findings should be interpreted as evidence of transcriptional regulation rather than direct changes in antioxidant enzyme activity or overall antioxidant capacity. Similar alterations in the Nrf2 signaling pathway have been described in poultry exposed to nutritional and oxidative stress (23, 27). In contrast, GPX1 expression showed greater variability among treatments and sampling times. At hatch, the IoT_36 group exhibited predominantly positive log_2_ fold-change values, whereas expression remained lower in the delayed-fed control group (Int_36). After feed deprivation, GPX1 expression became more heterogeneous, particularly in the Hydrogel®-supplemented groups, suggesting considerable individual variation in the antioxidant response. Such variability is biologically plausible because GPX1 represents a downstream component of the antioxidant defense system whose expression is influenced not only by Nrf2 signaling but also by tissue-specific metabolic activity and the local redox environment (46, 47).

Clear tissue-specific differences were observed between the jejunum and the liver. In the jejunum, expression of antioxidant-related genes generally remained closer to or above baseline after feed deprivation, suggesting activation of local adaptive mechanisms. This observation is consistent with the central role of the intestinal mucosa in early post-hatch adaptation, as the intestine is the first organ to encounter nutritional stress and rapidly adjusts its metabolic and antioxidant functions to preserve epithelial integrity (2, 8). Conversely, hepatic Nrf2 expression tended to decline in several delayed-fed groups, which may indicate a reduced capacity for systemic antioxidant regulation during prolonged nutritional challenge. Similar tissue-specific differences between intestinal and hepatic antioxidant responses have previously been described in poultry, reflecting the distinct physiological functions of these organs during metabolic adaptation (21, 44). The molecular findings were in good agreement with the performance results. Birds subjected to delayed access to feed showed reduced body weight and average daily gain during the early rearing period, indicating that the transcriptional alterations observed in antioxidant-related genes were accompanied by measurable physiological consequences. Although partial compensatory growth occurred later, growth performance did not fully recover in all delayed-fed groups, suggesting that early nutritional restriction may induce lasting metabolic changes. These observations are consistent with previous reports demonstrating that the first hours after hatch represent a critical developmental window in which nutritional management influences subsequent growth and metabolic programming (3, 22).

The in ovo administration of threonine appeared to exert its greatest influence during the immediate post-hatch period. The higher expression of antioxidant-related genes observed at hatch may indicate an early activation of protective pathways, which is consistent with the known physiological roles of threonine in mucin synthesis, intestinal barrier development, and immune function (13, 14). Nevertheless, these transcriptional differences became less evident following feed deprivation, particularly in the liver, suggesting that prolonged nutritional stress may outweigh the beneficial effects of prenatal nutritional supplementation. Hydrogel® supplementation also only provided limited benefits during the fasting period. Although gel supplementation supplies water and readily available nutrients that may support hydration and short-term energy balance, it was insufficient to restore growth performance or antioxidant responses to the level of immediately fed birds. This finding suggests that Hydrogel® cannot fully substitute for complete feed during the critical post-hatch transition, when nutrients are required not only to maintain energy metabolism but also to stimulate gastrointestinal development and metabolic adaptation (3, 22). Therefore, while Hydrogel® may represent a supportive management strategy under commercial conditions, immediate access to complete feed remains the most effective approach for minimizing the adverse consequences of delayed feeding.

The histological observations further complemented the physiological and molecular findings. Significant temporal changes were detected in hepatic lipid accumulation, hydropic degeneration, and lymphocytic infiltration. Lipid accumulation was most pronounced immediately after hatch, declined following feed deprivation, and partially increased again by Day 21, whereas inflammatory cell infiltration and hydropic degeneration became more evident after fasting and remained elevated in several delayed-fed groups. These alterations are consistent with the metabolic adjustments that occur in the liver during the transition from yolk-derived nutrients to exogenous feeding and may reflect the combined effects of oxidative stress and altered lipid metabolism. Similar relationships between oxidative stress, hepatic inflammation, and lipid metabolic disturbances have been described in ducks and other poultry species subjected to nutritional or metabolic stress (29).

The association observed between Nrf2 expression and hepatic lipid accumulation also supports the increasingly recognized role of the Nrf2 pathway beyond antioxidant defense. In addition to regulating cellular redox homeostasis, Nrf2 contributes to lipid metabolism, inflammatory signaling, and metabolic adaptation in the liver (27, 28). Although causality cannot be inferred from the present data, the combined molecular and histological findings suggest that early nutritional stress may induce persistent hepatic adaptations involving both oxidative and metabolic regulatory pathways. Considering the naturally active lipid metabolism of ducks compared with other poultry species, these birds may be particularly sensitive to disturbances occurring during the immediate post-hatch period.

## 5 Conclusion

The present study demonstrated that delayed access to feed during the first 36 h post-hatch induced substantial physiological alterations in ducks, including impaired early growth, oxidative imbalance, modified antioxidant-related gene expression, and histopathological changes in the liver. Delayed-fed birds exhibited reduced live weight, altered FRAP and MDA values, and treatment-dependent changes in GPX1 and Nrf2 expression, indicating that early nutritional stress affected both systemic and tissue-specific antioxidant responses. *In ovo* threonine administration and Hydrogel® supplementation influenced several antioxidant and molecular parameters during the immediate post-hatch period, particularly in relation to antioxidant activity and early gene expression patterns. However, these interventions were not sufficient to fully compensate for the adverse effects associated with delayed feeding. Histological findings further confirmed that early nutritional deprivation induced persistent hepatic alterations, including increased hydropic degeneration and inflammatory cell infiltration. Overall, the results emphasize the biological importance of immediate post-hatch nutrient availability for maintaining metabolic stability and supporting optimal early development in ducks. Although supportive nutritional strategies may contribute to certain adaptive responses, minimizing the delay between hatch and first feed access remains the most effective approach for reducing oxidative and physiological stress during the early post-hatch period.

## Data Availability

The raw data supporting the conclusions of this article will be made available by the authors, without undue reservation, upon reasonable request.
